# Effectiveness and safety of magnetic resonance–guided unilateral focused ultrasound subthalamotomy for Parkinson’s disease: a systematic review and meta-analysis of prospective studies

**DOI:** 10.3389/fnins.2025.1693035

**Published:** 2025-12-01

**Authors:** Bo Cheng, Zhang-hong Luo, Xiao Xiao, Cheng-fa Che, Tao Zhu, Shu-shan Zhang

**Affiliations:** 1 Department of Neurology, Affiliated Hospital of North Sichuan Medical College, Nanchong, China; 2 Department of Preventive Medicine, North Sichuan Medical College, Nanchong, China

**Keywords:** Parkinson’s disease, MRgFUS, subthalamotomy, treatment, meta-analysis

## Abstract

**Background:**

Patients with asymmetric, medication-refractory Parkinson’s disease (PD) often continue to experience disabling motor symptoms despite optimized pharmacological management. Magnetic resonance–guided focused ultrasound subthalamotomy (FUS-STN) has recently emerged as a promising, non-invasive alternative for improving motor function. However, its overall clinical efficacy and long-term safety remain the subject of active investigation.

**Methods:**

We systematically searched PubMed, Cochrane Library, Embase, Web of Science, and ClinicalTrials.gov from their inception to 30 November 2024. Prospective studies that assessed unilateral FUS-STN in patients with PD were included. Data were pooled using RevMan 5.3 for mean differences (MD) with 95% confidence intervals (CIs).

**Results:**

Four prospective studies (*n* = 69) were included. Unilateral FUS-STN significantly reduced the Movement Disorders Society-Unified Parkinson’s Disease Rating Scale (MDS-UPDRS) Part III scores for the treated hemibody in both the off-medication [MD = −11.01, 95% CI (−12.23, −9.80), *p* < 0.001] and on-medication states [MD = −6.51, 95% CI (−7.57, −5.42), *p* < 0.001]. The MDS-UPDRS II (MD = −3.05, *p* < 0.01) and 39-item Parkinson’s disease questionnaire summary index (PDQ-39SI) scores (MD = −6.99, *p* < 0.01) also improved. Levodopa equivalent daily dose (LEDD) was reduced in the short term (MD = −149.5 mg, *p* < 0.001), although it was attenuated at 12 months (*p* = 0.09). No significant improvement was observed in MDS-UPDRS IV scores (MD = −3.29, *p* = 0.64). In all included studies, adverse events (AEs) were frequent during and after the procedure, such as postoperative gait and speech disturbance, facial asymmetry, and dyskinesia. However, the majority of AEs were resolved during the 6–12 month follow-up period.

**Conclusion:**

Unilateral FUS-STN may offer symptomatic benefits and a general safety profile in selected patients with asymmetric PD. Future investigations should emphasize large-scale, longitudinal, multicenter, and symptom-specific randomized controlled trials to assess the long-term benefits and risks of unilateral FUS-STN in PD patients.

**Systematic review registration:**

https://www.crd.york.ac.uk/PROSPERO/view/CRD420251002754, identifier PROSPERO (CRD420251002754).

## 1 Introduction

Parkinson’s disease (PD) is the second most common neurodegenerative disorder, affecting over six million people worldwide, and its prevalence has more than doubled over the past three decades (Collaborators GN, 2019; Tolosa et al., 2021; Ben-Shlomo et al., 2024). Clinically, PD is characterized by various motor and non-motor features. The principal motor symptoms include tremor, rigidity, bradykinesia, and postural instability and gait disturbance (PIGD), each showing variable responsiveness to dopaminergic pharmacotherapy (Jankovic, 2008). This heterogeneity contributes to the diverse clinical trajectories and treatment challenges associated with PD management. Pharmacological treatment remains the first-line approach for managing motor dysfunction in patients with PD (Foltynie et al., 2024). Nevertheless, as the disease progresses, the therapeutic response often wanes, and patients develop motor fluctuations and dyskinesia that are difficult to control. When optimized pharmacotherapy fails to adequately control symptoms, deep brain stimulation (DBS) of the subthalamic nucleus (STN) or globus pallidus pars internus (GPi) by modulating the basal ganglia circuitry is considered the gold standard surgical intervention. DBS substantially improves tremor and bradykinesia; however, its efficacy in alleviating axial motor features, particularly PIGD, remains suboptimal, resulting in residual functional disability in a subset of patients (Krack et al., 2003).

Ablative functional neurosurgery has been used for decades to treat movement disorders (Hariz and Hariz, 2013). Emerging as a paradigm-shifting intervention, magnetic resonance–guided focused ultrasound (MRgFUS) enables precise non-invasive ablation of deep brain targets under real-time imaging feedback. Unlike DBS, MRgFUS requires neither craniotomy nor implanted hardware, offering distinct advantages for patients with contraindications to general anesthesia (Zhang et al., 2022). These technical advantages suggest that MRgFUS is a potential alternative to conventional second-line therapies for tremor-dominant PD (TDPD) and essential tremor refractory to pharmacotherapy (Natera-Villalba et al., 2024). Currently, the principal therapeutic targets include the GPi for the control of dyskinesia, the ventral intermediate nucleus (VIM) for tremor suppression, and the STN for comprehensive management of motor symptoms. Moreover, other structures, such as the pallido-thalamic tract (PTT) and cerebello-thalamic tract (CTT), may also serve as potential effective targets (Verhagen Metman et al., 2024; Stocchi et al., 2024). MRgFUS has shown significant benefits in reducing both on- and off-medication motor impairment scores in patients with PD (Krishna et al., 2023; Martínez-Fernández et al., 2020, 2024; Gallay et al., 2019; Bond et al., 2017). Thus, unilateral MRgFUS in a high-intensity modality targeting the VIM for TDPD received FDA approval in 2018. Subsequently, unilateral MRgFUS-GPi for PD dyskinesia was approved in 2021 (Verhagen Metman et al., 2024; Stocchi et al., 2024).

Recent investigations have examined the therapeutic potential of unilateral MRgFUS targeting the STN in PD patients (Martínez-Fernández et al., 2018, 2020, 2024; Guida et al., 2024; Armengou-Garcia et al., 2024). A pivotal randomized controlled trial (RCT) (Martínez-Fernández et al., 2020) involving 40 patients with markedly asymmetric PD demonstrated that unilateral FUS-STN produced significant motor improvement, with a reduction of 8.1 points on the Movement Disorders Society-Unified Parkinson’s Disease Rating Scale (MDS-UPDRS) part III. However, the study also raised concerns regarding the relatively high incidence of adverse events (AEs), including gait disturbances and dysarthria (Perlmutter and Ushe, 2020). In addition to individual trials, several systematic reviews and meta-analyses have assessed the clinical efficacy and safety of MRgFUS for PD, encompassing various targets such as VIM, GPi, PTT, and STN (Liang et al., 2025; Balduino de Souza et al., 2025; Monteiro et al., 2024; Abbas et al., 2024; Tian et al., 2023; Ge et al., 2021). For example, Tian et al. reported notable motor improvements accompanied by an overall favorable safety profile across these targets (Tian et al., 2023). Similarly, Ge et al. observed substantial reductions in tremor severity and improvements in daily activity performance in studies focusing on tremor-dominant PD phenotypes (Ge et al., 2021). Moreover, a network meta-analysis comparing FUS-STN with STN- and GPi-DBS revealed comparable efficacy in both motor and quality-of-life outcomes (Liang et al., 2025). Consistently, Balduino de Souza et al. summarized evidence from multiple RCTs and concluded that MRgFUS exhibits a generally safe profile, with the majority of adverse effects being transient (Balduino de Souza et al., 2025).

Despite these promising findings, the current evidence remains relatively fragmented. A previous meta-analysis that evaluated the efficacy of FUS-STN may have been limited in determining its specific therapeutic benefits for PD, as it included studies involving heterogeneous targets. Therefore, this study focuses exclusively on unilateral FUS-STN, synthesizing evidence from prospective studies, including RCTs published through late 2024, to provide an updated and comprehensive assessment of its efficacy in improving motor function and activities of daily living, as well as its safety in patients with asymmetric, medication-refractory PD.

## 2 Methods

This meta-analysis was conducted following the (Cochrane, 2024) and the Preferred Reporting Items for Systematic Reviews and Meta-Analysis (PRISMA) guidelines (PROSPERO: CRD420251002754) (Moher et al., 2009).

### 2.1 Search and selection strategy

A systematic search was conducted across several databases, including MEDLINE (PubMed), Cochrane Library, Embase, Web of Science, and ClinicalTrials.gov, from their inception to 30 November 2024. The search strategy included the following terms: (“focused ultrasound subthalamotomy” OR “HiFU subthalamotomy” OR “MRgFUS subthalamotomy” OR “MRgFUS subthalamic nucleus” OR “MRgFUS STN” OR “MRgFUS ablation” OR “subthalamic nucleus focused ultrasound ablation”) AND (“Parkinso*” OR “PD”). References from the relevant reviews were manually searched to identify additional qualifying studies. Deduplication was performed using the NoteExpress software. The studies included in our analysis met the following eligibility criteria: (1) studies involving PD patients treated with unilateral FUS-STN; (2) assessment of clinical endpoints related to efficacy and safety; and (3) randomized controlled trials (RCTs) or other prospective study designs. The exclusion criteria were as follows: (1) non-English publications; (2) case reports, reviews, and conference abstracts; and (3) studies with unreliable or unconvertible data. For studies involving overlapping groups of PD patients, we selected publications with the largest sample sizes and motor symptom scale scores as the primary outcomes for inclusion in this meta-analysis.

### 2.2 Selection process

Two authors (XX and Z-hL) independently screened the titles and abstracts to identify eligible studies. The full texts of potentially relevant studies were independently assessed by two other authors (BC and Z-hL). Disagreements regarding the study eligibility were resolved through consensus discussions with two additional authors (TZ and S-sZ).

### 2.3 Data extraction

Two investigators (BC and Z-hL) independently extracted the data using a standardized form with duplicate extraction to ensure accuracy. Discrepancies were resolved through consensus discussions mediated by a third author (TZ). Characteristics were extracted in categories such as study design, patient age, number of patients, duration of PD, follow-up period, baseline MDS-UPDRS III scores, baseline levodopa equivalent daily dose (LEDD) (Tomlinson et al., 2010), and primary clinical endpoints. Outcome data were extracted, which included the number of AEs during and post-procedure, as well as clinical outcomes presented as mean and standard deviation (SD). In one study, data on efficacy outcomes were listed as median (interquartile range) (Martínez-Fernández et al., 2024). We, therefore, applied validated methods to estimate means and SD from median-based data after evaluating skewness (Shi et al., 2023; Wan et al., 2014; Luo et al., 2018). The efficacy outcomes included MDS-UPDRS III scores (range, 0–44; higher values indicate more severe motor impairment) (Goetz et al., 2008) for the treated side in both on- and off-medication states, MDS-UPDRS II (range, 0–52; higher values denote greater disability in activities of daily living), MDS-UPDRS IV (range, 0–24; higher values reflect more frequent and disabling motor complications) (Goetz et al., 2008), the 39-item Parkinson’s Disease Questionnaire summary index [(PDQ-39SI); range, 0–100, with higher values representing poorer quality of life] (Peto et al., 1995), and LEDD.

### 2.4 Quality assessment

After full-text screening, the included studies were independently evaluated for quality by two authors (BC and Z-hL), and the final judgment was made by consensus. The Cochrane Risk of Bias in Non-randomized Studies of Interventions (ROBINS-I) tool was used to assess the non-randomized studies (Sterne et al., 2016). Meanwhile, the RoB 2 tool (Cochrane Methods) was used to assess randomized controlled studies (Sterne et al., 2019).

### 2.5 Statistical analysis

The meta-analysis was performed in accordance with the (Cochrane, 2024). Statistical analyses were conducted using Review Manager (RevMan) version 5.3 (Cochrane Collaboration). For continuous variables, pooled mean differences (MD) with corresponding 95% confidence intervals (CIs) were calculated. A random-effects model was used when significant heterogeneity was detected (*I*^2^ > 50% or *p* < 0.10, Cochran’s *Q* test) (Higgins and Thompson, 2002), as it accounts for both within- and between-study variability. Otherwise, a fixed-effects model was applied (Cochrane, 2024). Publication bias was visually assessed using funnel plots, recognizing that funnel plots are unreliable when fewer than ten studies are included. A *p* value of <0.05 was considered statistically significant.

## 3 Results

### 3.1 Study selection

**Figure 1:**
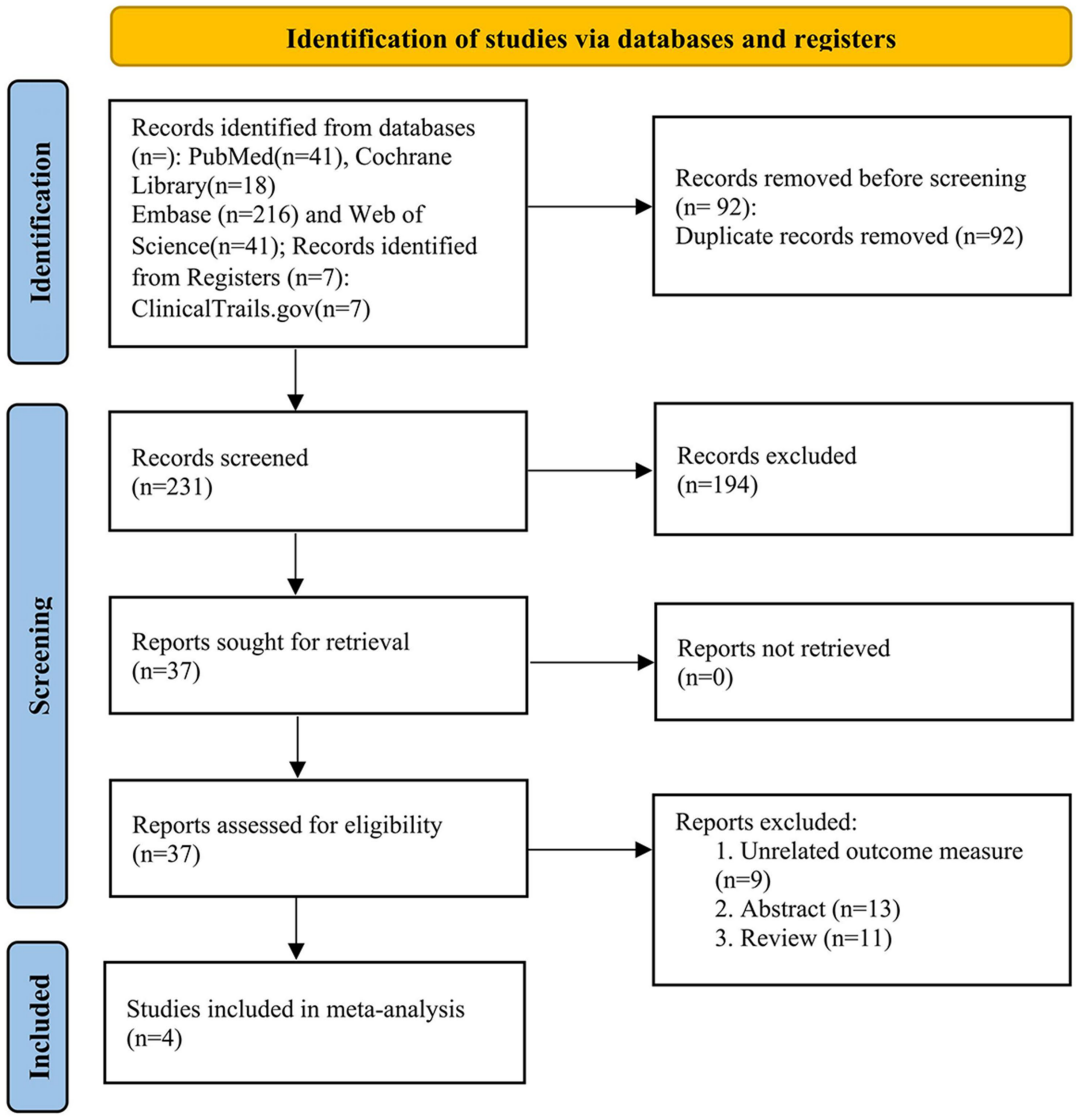
The PRISMA flowchart of the literature search and study selection process.

The search strategy identified 323 records. After eliminating duplicates, the total number was reduced to 231. Following screening of titles and abstracts, 37 articles met the criteria for further full-text review, of which 33 were excluded. Notably, one study (Martínez-Fernández et al., 2023) was excluded because its participants overlapped with those of two included studies (NCT02912871/03454425) (Martínez-Fernández et al., 2018, 2020, 2024). Ultimately, four studies (Martínez-Fernández et al., 2018, 2020; Armengou-Garcia et al., 2024) involving 69 PD patients were included in the primary meta-analysis. The Preferred Reporting Items for Systematic reviews and Meta-Analyses (PRISMA) flowchart of the literature search and study selection process is shown in Figure 1.

### 3.2 Quality assessment and publication bias

The included studies were assessed by two independent investigators (BC and S-sZ). One RCT had some concerns about the risk of bias, as assessed by the RoB 2 tool (Sterne et al., 2019). Of the remaining studies, two were rated as moderate risk and one as serious risk using the ROBINS-I (Sterne et al., 2016). The risk of bias analysis is summarized in Supplementary Figures 1A,B. Visual inspection of the funnel plots (Supplementary Figures 2–7) revealed no substantial evidence of publication bias for the means and SD.

### 3.3 Study characteristics

The median age of patients was more than 50 years, and the median disease duration ranged from 3 to 7.5 years. The number of PD patients included ranged from 10 to 40. Each study had a maximum follow-up period of 12 months; however, one study extended the follow-up period to 12 months using post-hoc analysis. All studies assessed motor features both in the on- and off-medication states at baseline, except for one study (Armengou-Garcia et al., 2024), which reported only on-medication MDS-UPDRS III scores. The primary efficacy outcome of the included studies was the MDS-UPDRS III score for the treated hemibody in the off-medication state. Baseline LEDD ranged from 560.0 (median) to 881.7 (mean) mg. The characteristics of the included studies are shown in Table 1.

**Table 1:** Characteristics of included studies.

Study	Design	Age (year)	No. of patients	PD duration or duration since diagnosis (year)	Follow-up	Baseline MDS-UPDRS III scores, on-state/off-medication state	Baseline LEDD, mg	Primary outcomes
Martínez-Fernández et al. (2018)	Prospective, open-label case series	59.5 ± 10.1	10 asymmetrical patients with PD	6.3 ± 2.5	At 1,3, 6, and 12 months	21.5 ± 6.3/32.7 ± 5.4	732.7 ± 346.4	Efficacy outcomes: the mean MDS–UPDRS III score on the treated side improved by 53% from baseline to 6 months in the off-medication state [16.6 (SD 2.9) vs. 7.5 (3.9)] and by 47% in the on-medication state [11.9 (3.1) vs. 5.8 (3.5)]; Safety outcomes: reported AEs at 6 months
Martínez-Fernández et al. (2020)	Prospective, randomized, controlled, double-blind clinical trial	56.6 ± 9.3^a^	40 asymmetrical patients with PD (27 receiving real FUS-STN treatment and 13 a sham procedure)	5.6 ± 2.5^a^	At 2, 4, and 12 months	26.9 ± 6.7/39.9 ± 9.7^a^	729.7 ± 328.3^a^	Efficacy outcomes: at 4 months, the difference in the mean MDS-UPDRS III score on the treated side between the active treatment group and the control group was 8.1 points (95% CI, 6.0 to 10.3; *p* < 0.001) in the off-medication state. Safety outcomes: the incidence and severity of AEs at 4 months
						25.1 ± 8.1/40.1 ± 8.1^b^	881.7 ± 407.9^b^	
		58.1 ± 8.8^b^		7.3 ± 3.8^b^				
Armengou-Garcia et al. (2024)	Prospective, open-label case series	62.5 (56.8–66.0)	20 asymmetrical patients with PD	7.5 (3.8–9.2)	At 1, 3, 6, and 12 months	NA/32.0 (27.0–40.2)	585.0 (450.0–812.5)	Efficacy outcomes: the mean MDS-UPDRS III score on the treated side decreased from 17.5 (SD 5.0) at baseline to 5.0 (3.7) at 12 months in the off-medication state; the mean difference was 12.5 points (*p* < 0.001). Safety outcomes: the frequency and severity of AEs at 6 months
Martínez-Fernández et al. (2024)	Prospective, open-label case series	52.0 (49.8–55.3)	12 asymmetrical patients with PD	3.0 (2.1–3.9)	At 3, 6, and 12 months	18.0 (12.7–19.5)/26.5 (23.2–32.2)	560.0 (498.7–668.7)	Safety outcomes: treatment-related AEs at 6 months

Data are listed as mean ± standard deviation or median (interquartile range).

a Group receiving real FUS-STN treatment.

b Group receiving a sham procedure.

AEs, adverse events; CI, confidence interval; FUS-STN, unilateral focused ultrasound subthalamotomy; LEDD, levodopa equivalent daily dose; MDS-UPDRS III, the Movement Disorders Society-Unified Parkinson’s Disease Rating Scale, Part III; PD, Parkinson’s Disease; SD, standard deviation.

### 3.4 Outcomes of effectiveness

#### 3.4.1 MDS-UPDRS III scores for treated side

**Figure 2:**
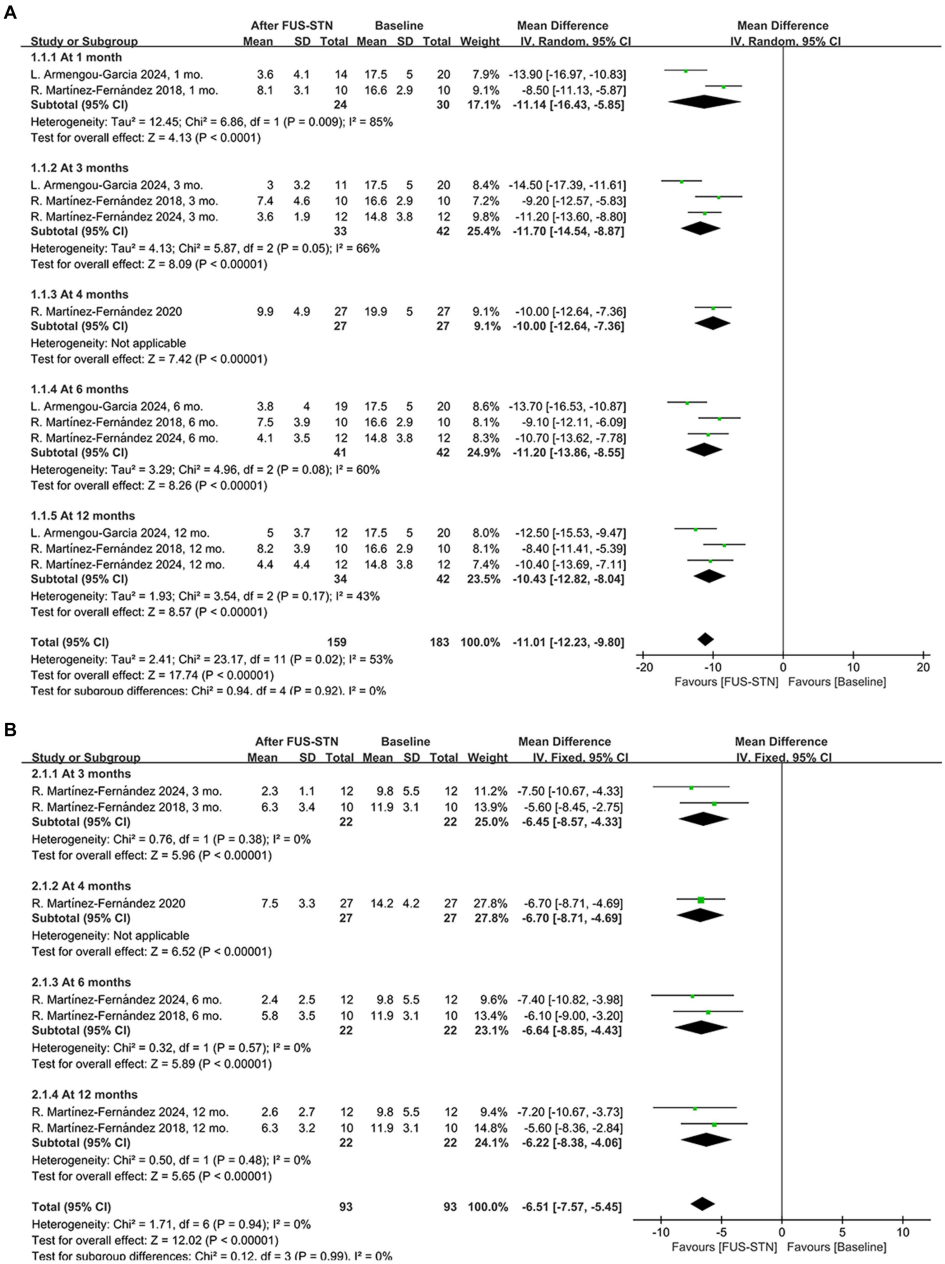
Forest plot of pooled MDS-UPDRS III scores for the treated hemibody following unilateral FUS-STN. **(A)** Off-medication state at 1, 3, 4, 6, and 12 months; **(B)** On-medication state at 3, 4, 6, and 12 months.

A total of 4 studies (*n* = 69 patients with PD) evaluated the efficacy of unilateral FUS-STN, primarily based on the MDS-UPDRS III scores of the treated hemibody. Among these, three studies assessed outcomes under both off- and on-medication conditions. Compared with baseline, unilateral FUS-STN produced significant reductions in MDS-UPDRS III scores in both conditions. Compared to baseline, unilateral FUS-STN significantly reduced MDS-UPDRS III scores in both states [off-medication: MD = −11.01, 95% CI (−12.23, −9.80), *p* < 0.0001, *I*^2^ = 53%; on-medication: MD = −6.51, 95% CI (−7.57, −5.45), *p* < 0.0001, *I*^2^ = 0%; Figures 2A,B].

**Figure 3:**
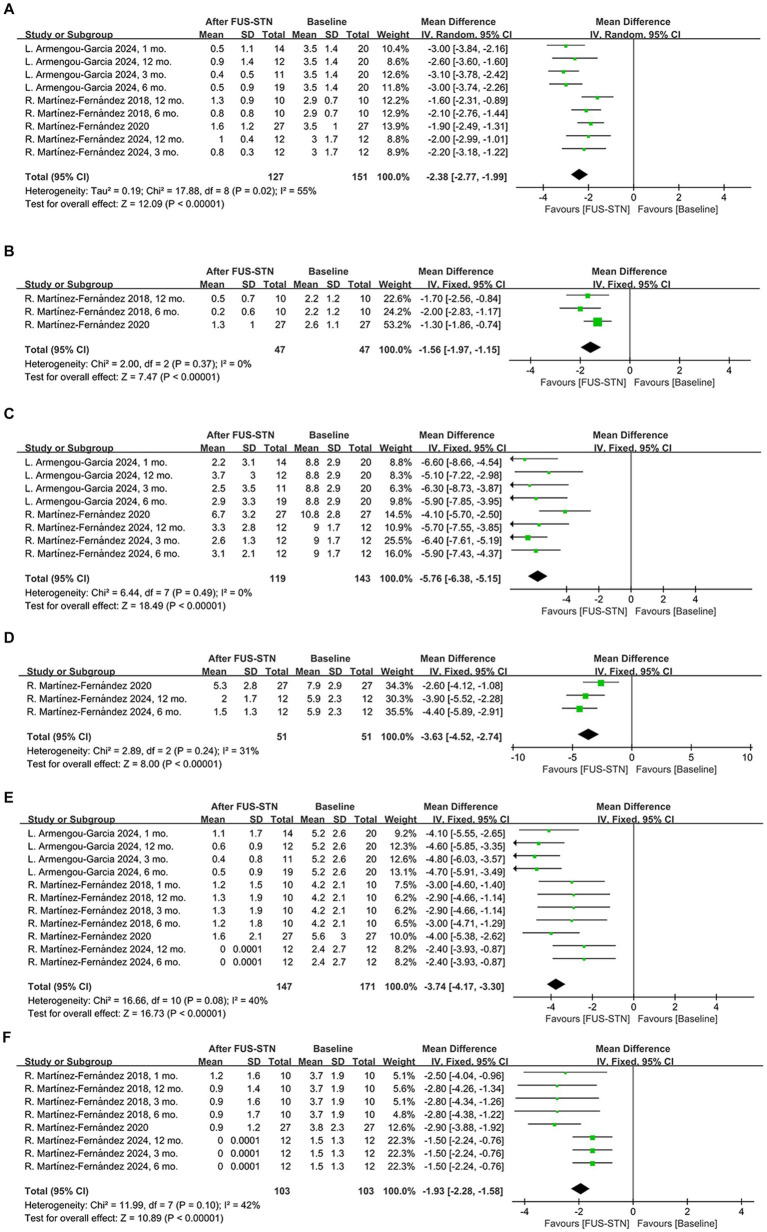
Forest plot of pooled MDS-UPDRS III subitem scores for the treated hemibody following unilateral FUS-STN. **(A)** Rigidity in the off-medication state; **(B)** rigidity in the on-medication state; **(C)** bradykinesia in the off-medication state; **(D)** bradykinesia in the on-medication state; **(E)** tremor in the off-medication state; and **(F)** tremor in the on-medication state.

Subgroup analysis of the MDS-UPDRS III subitems further revealed significant improvements in both the off- and on-medication states for rigidity [off-medication: MD = −2.38, 95% CI (−2.77, −1.99), *p* < 0.0001, *I*^2^ = 53%; on-medication: MD = −1.56, 95% CI (−1.97, −1.15), *p* < 0.0001, *I*^2^ = 0%, Figures 3A,B], bradykinesia [off-medication: MD = −5.76, 95% CI (−6.38, −5.15), *p* < 0.0001, *I*^2^ = 0%; on-medication: MD = −3.63, 95% CI (−4.52, −2.74), *p* < 0.0001, *I*^2^ = 31%, Figures 3C,D], and tremor [off-medication: MD = −3.74, 95% CI (−4.17, −3.30), *p* < 0.0001, *I*^2^ = 40%; on-medication: MD = −1.93, 95% CI (−2.28, −1.58), *p* < 0.0001, *I*^2^ = 42%; Figures 3E,F]. To evaluate the durability of the therapeutic effects, subgroup analyses stratified by follow-up duration were conducted. In the off-medication state, unilateral FUS-STN brought about significant improvement of the MDS-UPDRS III score from baseline to several follow-ups [at 1 month: MD = −11.14, 95% CI (−16.43, −5.85), *p* < 0.0001, *I*^2^ = 85%; at 3 months: MD = −11.70, 95% CI (−14.54, −8.87), *p* < 0.0001, *I*^2^ = 66%; at 4 months: MD = −10.0, 95% CI (−12.64, −7.36), *p* < 0.0001; at 6 months: MD = −11.20, 95% CI (−13.86, −8.55), *p* < 0.0001, *I*^2^ = 60%; at 12 months: MD = −10.43, 95% CI (−12.82, −8.04), *p* < 0.0001, *I*^2^ = 43%; Figure 2A]. Similarly, significant improvements from baseline were also observed in the on-medication state [at 3 months: MD = −6.45, 95% CI (−8.57, −4.33), *p* < 0.0001, *I*^2^ = 0%; at 4 months: MD = −6.70, 95% CI (−8.71, −4.69), *p* < 0.0001; at 6 months: MD = −6.64, 95% CI (−8.85, −4.43), *p* < 0.0001, *I*^2^ = 0%; at 12 months: MD = −6.22, 95% CI (−8.38, −4.06), *p* < 0.0001, *I*^2^ = 0%; Figure 2B].

#### 3.4.2 MDS-UPDRS II scores

**Figure 4:**
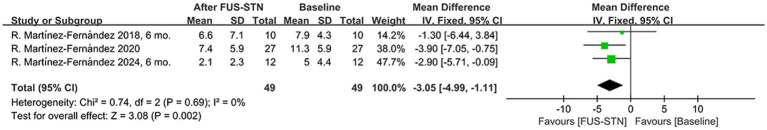
Forest plot of pooled MDS-UPDRSII scores following unilateral FUS-STN.

Pooled MDS-UPDRS II scores (reflecting activities of daily living), involving 49 patients with PD, showed a significant reduction in unilateral FUS-STN post-procedure compared to baseline [MD = −3.05, 95% CI (−4.99, −1.11), *p* = 0.002, *I*^2^ = 0%; Figure 4].

#### 3.4.3 MDS-UPDRS IV scores

**Figure 5:**
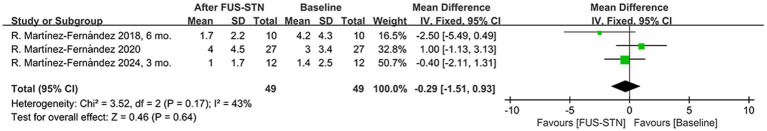
Forest plot of pooled MDS-UPDRSIV scores following unilateral FUS-STN.

Motor complications pose challenges to PD interventions, particularly in pharmacological therapy. Pooled MDS-UPDRS IV scores from three studies (*n* = 49 patients with PD) indicated that unilateral FUS-STN did not significantly alleviate motor complications [MD = −3.29, 95% CI (−1.51, 0.93), *p* = 0.64, *I*^2^ = 43%; Figure 5].

#### 3.4.4 PDQ-39SI scores

**Figure 6:**
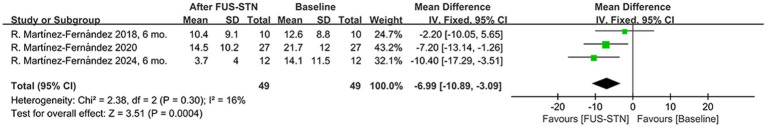
Forest plot of pooled PDQ-39SI scores following unilateral FUS-STN.

PDQ-39SI scores were used to assess the impact of unilateral FUS-STN on quality of life in 49 patients with PD. Pooled scores showed a significant decrease post-procedure compared to baseline [MD = −6.99, 95% CI (−10.89, −3.09), *p* = 0.0004, *I*^2^ = 16%; Figure 6].

#### 3.4.5 LEDD

**Figure 7:**
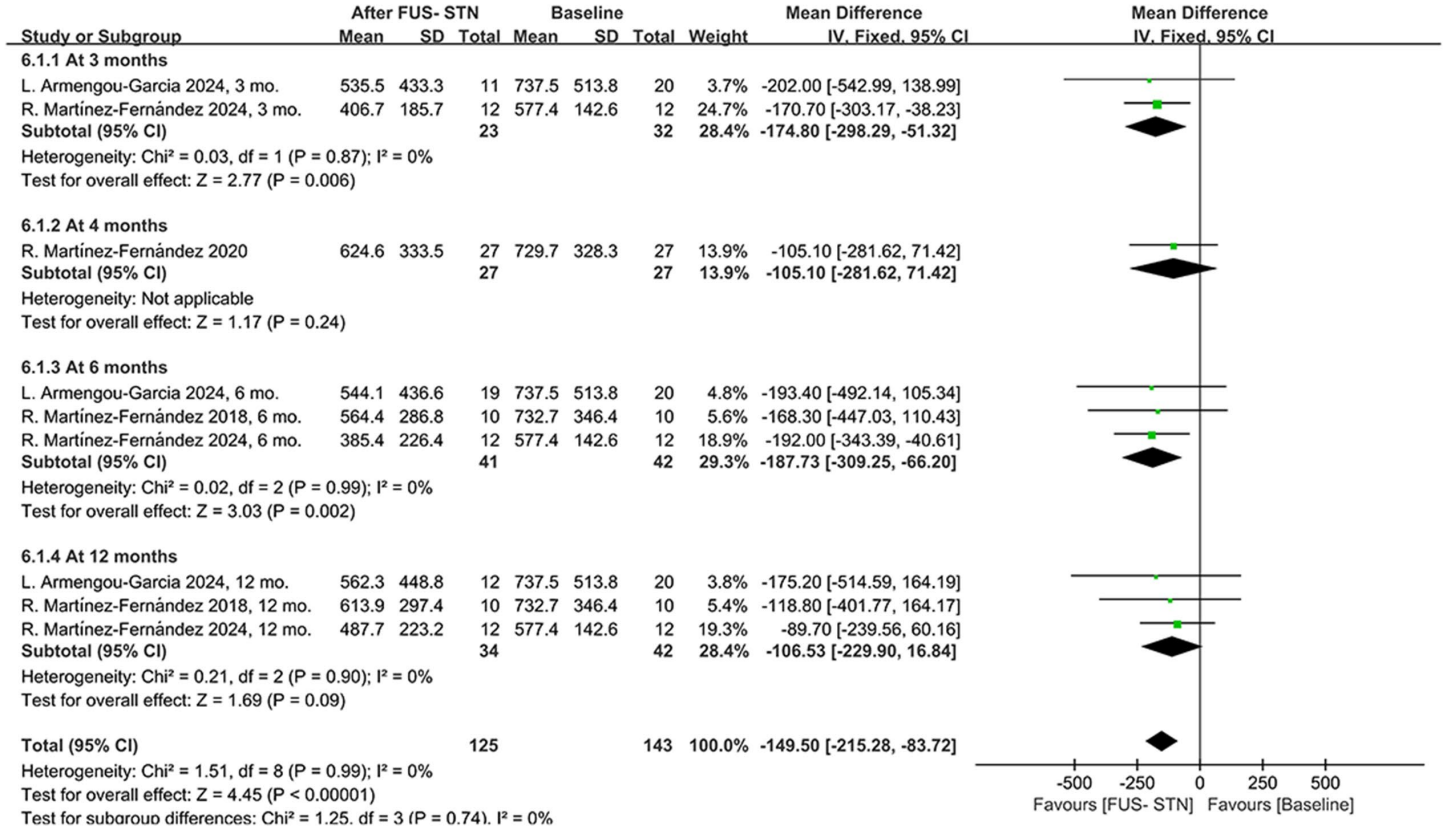
Forest plot of pooled LEDD following unilateral FUS-STN at 3, 4, 6, and 12 months.

Following unilateral FUS-STN in patients with PD, LEDD adjustments were observed, suggesting potential therapeutic efficacy. A pooled analysis of four studies demonstrated a significant overall LEDD reduction during follow-up [MD = −149.50, 95% CI (−215.28, −83.72), *p* < 0.0001, *I*^2^ = 0%; Figure 7]. Subgroup analyses stratified by follow-up duration revealed heterogeneous outcomes. Specifically, LEDD significantly decreased at 3 and 6 months post-procedure compared to baseline [at 3 months: MD = –174.80, 95% CI (−298.29, −51.32), *p* = 0.0006, *I*^2^ = 0%; at 6 months: MD = −187.73, 95% CI (−309.25, −66.20), *p* = 0.002, *I*^2^ = 0%]. However, the results from one RCT showed no significant difference in LEDD at 4 months post-procedure [MD = −105.10, 95% CI (−281.62, 71.42), *p* = 0.24]. Similarly, there was no significant reduction in LEDD at 12 months [MD = −106.53, 95% CI (−229.90, 16.84), *p* = 0.09, *I*^2^ = 0%].

### 3.5 Outcomes of safety

Adverse events (AEs) were monitored during and after the procedure in all included studies. The most common intraprocedural AEs included pin site pain, head tilting, elevated blood pressure, dizziness, discomfort (such as sensations of heat or pressure), and nausea. Postprocedural AEs such as gait disturbance, speech abnormalities, facial asymmetry, and dyskinesia were generally mild to moderate, with most AEs resolving during the follow-up period. Persistent AEs at the final follow-up (6–12 months) were reported in these studies. A summary of the AEs observed during and after the procedure is provided in Tables 2, 3.

**Table 2:** Summary of adverse events during and after the procedure.

Study	Adverse events during the procedure	Adverse events after the procedure
Martínez-Fernández et al. (2018)	Pin-site head pain (*n* = 6), high blood pressure (*n* = 5), nausea (*n* = 4), warm cranial sensation (*n* = 2), back pain (*n* = 2), and anxiety (*n* = 2)	Gait ataxia (*n* = 6), behavioral disinhibition (*n* = 2), facial palsy (*n* = 1), off-medication, upper limb dyskinesia (*n* = 1), new-onset, on-medication, upper limb dyskinesia (*n* = 1), subjective speech disturbance (*n* = 1), anxiety (*n* = 1), fatigue (*n* = 1) and weight gain (*n* = 2)
Martínez-Fernández et al. (2020)	Pin-site head pain (*n* = 16), head “tilting” (*n* = 13), dizziness (*n* = 13), head discomfort, such as “heat” or “pressure” (*n* = 11), nausea (*n* = 7), high blood pressure (*n* = 7), anxiety (*n* = 6), headache (*n* = 5), back or neck pain (*n* = 4), emesis (*n* = 1), right inner ear pain (*n* = 1) and fatigue (*n* = 1)	Speech disturbance (*n* = 15), gait disturbance (*n* = 13), off-medication, dyskinesia on the more affected side (*n* = 6), on-medication, new-onset dyskinesia on the more affected side (*n* = 6), weakness on the more affected side (*n* = 5), isolated facial asymmetry (*n* = 3), upper limb dysmetria (*n* = 2), weight gain (*n* = 2), impulsiveness (*n* = 1) and somnolence (*n* = 1)
Armengou-Garcia et al. (2024)	Dysarthria (*n* = 4), off-medication, *de novo* dyskinesia (*n* = 4), and facial asymmetry (*n* = 2)	Weight gain (*n* = 7), behavioral changes for hypomania (*n* = 6), facial asymmetry (*n* = 4), weakness (*n* = 3), gait disturbance for dystonic pseudo foot drop (*n* = 3), on-medication, de novo dyskinesia (*n* = 3), off-medication, de novo dyskinesia (*n* = 2), dysarthria (*n* = 2), paresthesia (*n* = 2) and confusional state (*n* = 1)
Martínez-Fernández et al. (2024)	Head “tilting” (*n* = 4), headache (*n* = 3), high blood pressure (*n* = 3), asymptomatic bradycardia (*n* = 2), vagal reaction during frame placement (*n* = 2), nausea (*n* = 1), emesis (*n* = 1), dizziness (*n* = 1), and head discomfort, such as “heat” or “pressure” (*n* = 1)	Weight gain (*n* = 7), isolated facial asymmetry (*n* = 6), unsteady gait, as reported by the patient (*n* = 5), on-medication, new-onset dyskinesia on the treated side (*n* = 4), subjective speech abnormalities (*n* = 2), foot dystonia (*n* = 2), off-medication, dyskinesia on the treated side (*n* = 1), pericranial hypoesthesia (*n* = 1), hiccup (*n* = 1) and cheerfulness (*n* = 1)

Data are presented as the number of patients (in brackets).

**Table 3:** Summary of short- and long-term adverse events.

Study	Short-term adverse events^a^	Long-term adverse events^b^
Martínez-Fernández et al. (2018)	Transient mild gait ataxia (*n* = 6) and facial asymmetry (*n* = 1)	Off-medication, upper limb dyskinesia (*n* = 1), new-onset, on-medication, upper limb dyskinesia (*n* = 1) and subjective speech disturbance (*n* = 1), anxiety (*n* = 1), fatigue (*n* = 1) and weight gain (*n* = 2)
Martínez-Fernández et al. (2020)	Weakness on the more affected side (*n* = 5), isolated facial asymmetry (*n* = 3), speech disturbance (*n* = 6), gait disturbance (*n* = 8) and somnolence (*n* = 1)	On-medication, new-onset dyskinesia on the more affected side (*n* = 2), weakness on the more affected side (*n* = 2), speech disturbance(n = 1), gait disturbance (*n* = 1) and weight gain(n = 2)
Armengou-Garcia et al. (2024)	Facial asymmetry (*n* = 4), weakness (*n* = 3), dysarthria (*n* = 2), gait disturbance for dystonic pseudo foot drop (*n* = 3) and confusional state (*n* = 1)	Weakness (*n* = 1), gait disturbance for dystonic pseudo foot drop (*n* = 1) and weight gain (*n* = 1)
Martínez-Fernández et al. (2024)	Isolated facial asymmetry (*n* = 6), subjective speech abnormalities (*n* = 2), contralateral weakness (*n* = 1), unsteady gait, as reported by the patient (*n* = 5), hiccup (*n* = 1) and cheerfulness (*n* = 1)	Isolated facial asymmetry (*n* = 1), subjective speech abnormalities (*n* = 1) and weight gain (*n* = 7)

Data are presented as the number of patients (in brackets).

a Adverse events at 24-h post-procedure.

b Adverse events at the last follow-up (6–12 months).

## 4 Discussion

This systematic review and meta-analysis provided updated evidence on the clinical efficacy and safety of unilateral FUS-STN in patients with asymmetric, medication-refractory PD. Across the included prospective studies, unilateral FUS-STN resulted in significant and sustained improvements in overall motor performance, daily functioning, and quality of life, as reflected by reductions in MDS-UPDRS III and II and PDQ-39SI scores. Importantly, these motor improvements were clinically meaningful in both off- and on-medication states and persisted for up to 6–12 months after treatment (Schrag et al., 2006). Subgroup analysis of the motor subcomponents revealed that rigidity, bradykinesia, and tremor improved significantly following FUS-STN, highlighting its broad therapeutic potential. However, no significant benefit was observed in improving motor complications, and its effects on gait and other axial motor symptoms remain uncertain. Further research is needed to clarify these aspects, which will continue to pose therapeutic challenges. Additionally, we found a significant reduction in the LEDD following unilateral FUS-STN compared to baseline, although the LEDD gradually returned to baseline levels by the 12-month follow-up. This trend may reflect the progressive nature of PD, particularly on the untreated side, necessitating the gradual escalation of dopaminergic therapy to address advancing disability. Collectively, these longitudinal data suggest that the therapeutic effects of unilateral FUS-STN may attenuate over time.

Safety remains a critical consideration for any ablative neurosurgical intervention. Across the included studies, intraoperative AEs, most commonly pin-site pain, dizziness, transient hypertension, and sensations of cranial heat or pressure, were generally self-limiting and manageable during the procedure (Martínez-Fernández et al., 2018, 2020, 2024; Armengou-Garcia et al., 2024). Mechanistically, discomfort during sonication is primarily attributed to the thermal effects of high-intensity ultrasound and frame fixation; however, dizziness may also arise from the activation of vestibular pathways/fibers adjacent to the STN and those traveling toward the VIM, as documented in both DBS and MRgFUS studies (Beylergil et al., 2024; Ciocca et al., 2024). Early postoperative AEs, including gait disturbance, dysarthria, weakness, facial asymmetry, and dyskinesia, were typically mild to moderate in severity and tended to improve over time, consistent with transient edema or microstructural changes around the lesion site (Tian et al., 2023). Regarding weight gain observed in some patients, it has been speculated that this may reflect improvements in motor function and subsequent reductions in energy expenditure. However, direct mechanistic evidence remains limited (Balduino de Souza et al., 2025; Tian et al., 2023). Although the reporting of AEs varied among the studies, most were mild to moderate in severity, supporting the notion that unilateral FUS-STN is generally safe and tolerated. A longer follow-up period is required to comprehensively assess the long-term safety and durability of this novel intervention. Notably, a recent extension study combining two previous trials (*n* = 32) demonstrated a 52.3% improvement in off-medication MDS-UPDRS III scores for the treated side at 3 years, accompanied by reductions across all motor subscores without severe or permanent AEs (Martínez-Fernández et al., 2023). Moreover, a recent controlled trial indicated that unilateral FUS-STN does not impair social cognition and may even enhance certain cognitive domains in patients with PD (Guida et al., 2024). These findings are encouraging but require further validation in larger cohorts. Future research should focus on identifying predictors of therapeutic response and individualizing treatment parameters based on patient-specific characteristics, such as clinical phenotypes and neuroimaging biomarkers, to optimize both efficacy and safety (Lin et al., 2022).

Our study has several limitations. First, only one RCT was included, whereas the remaining studies adopted an open-label prospective design. This limitation reduces the overall strength of the evidence. Second, the relatively small sample size (four studies) and short follow-up duration (≤12 months), with three studies conducted by the same research team, diminished the robustness and generalizability of the findings. Third, because of insufficient data, subgroup analyses based on patient characteristics could not be conducted. Furthermore, this meta-analysis did not provide quantitative estimates of the resolution or persistence of AEs. Finally, the limited number of studies precluded a robust assessment of publication bias.

Future research should aim to conduct larger, multicenter, long-term RCTs to assess the symptom-specific efficacy, durability of benefits, and neuropsychiatric safety of unilateral FUS-STN. It is also essential to further explore comparisons with other lesioning targets (e.g., GPi, VIM, PTT) and DBS outcomes, particularly focusing on the durability of the effects and bilateral disease progression. Longitudinal studies incorporating neuroimaging biomarkers are critical for optimizing treatment parameters for personalized therapy.

## 5 Conclusion

This meta-analysis reinforces the therapeutic potential of unilateral FUS-STN for asymmetric PD, demonstrating clinically meaningful improvements in motor performance and quality of life, along with manageable and predominantly transient AEs. Future studies should emphasize large-scale, longitudinal, multicenter, and symptom-specific RCTs to evaluate the long-term efficacy and safety of unilateral FUS-STN in patients with PD.

## Data Availability

The original contributions presented in the study are included in the article/Supplementary material, further inquiries can be directed to the corresponding author.

## Glossary

AEs: adverse events
CI: confidence interval
DBS: deep brain stimulation
FDA: Food and Drug Administration
FUS-STN: focused ultrasound subthalamotomy
GPi: globus pallidus pars interna
LEDD: levodopa equivalent daily dose
MAO-B: monoamine oxidase B
MD: mean difference
MDS-UPDRS: Movement Disorders Society-Unified Parkinson’s Disease Rating Scale
MRI: magnetic resonance imaging
MRgFUS: magnetic resonance–guided focused ultrasound
MRgHiFU: magnetic resonance–guided high-intensity focused ultrasound
PD: Parkinson’s disease
PDQ-39SI: 39-item Parkinson’s disease questionnaire summary index
PRISMA: Preferred Reporting Items for Systematic Reviews and Meta-Analysis
RCT: randomized controlled trial
RoB 2: Risk of Bias 2
ROBINS-I: Risk of Bias in Non-randomized Studies of Interventions
SD: standard deviation
STN: subthalamic nucleus
TDPD: tremor-dominant Parkinson’s disease
VIM: ventral intermediate nucleus
