# Supplementary material for: Effectiveness and safety of magnetic resonance–guided unilateral focused ultrasound subthalamotomy for Parkinson’s disease: a systematic review and meta-analysis of prospective studies
Source: Front Neurosci. 2025 Dec 1;19:1693035. doi: 10.3389/fnins.2025.1693035 (PMC12702895; doi:10.3389/fnins.2025.1693035)
Supplement: Supplementary file 1 [file Data_Sheet_1.docx]

Supplementary Material

## Supplementary Figures

(A)


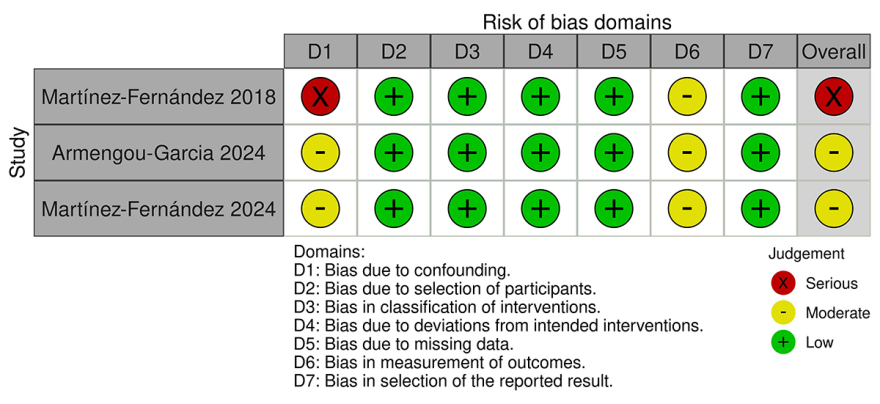


(B)


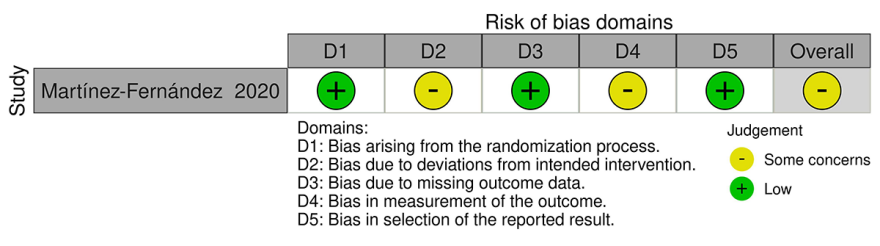


**Supplementary** **Figure 1** Summary of risk of bias analysis. (A) ROBINS‐I tool for non‐randomised studies; (B) RoB 2 for randomized controlled study

(A)


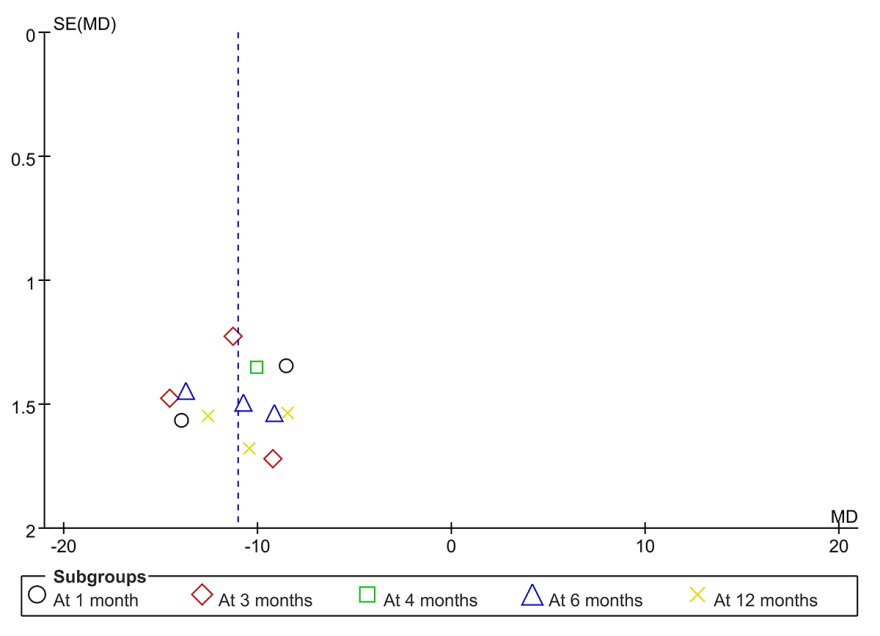


(B)


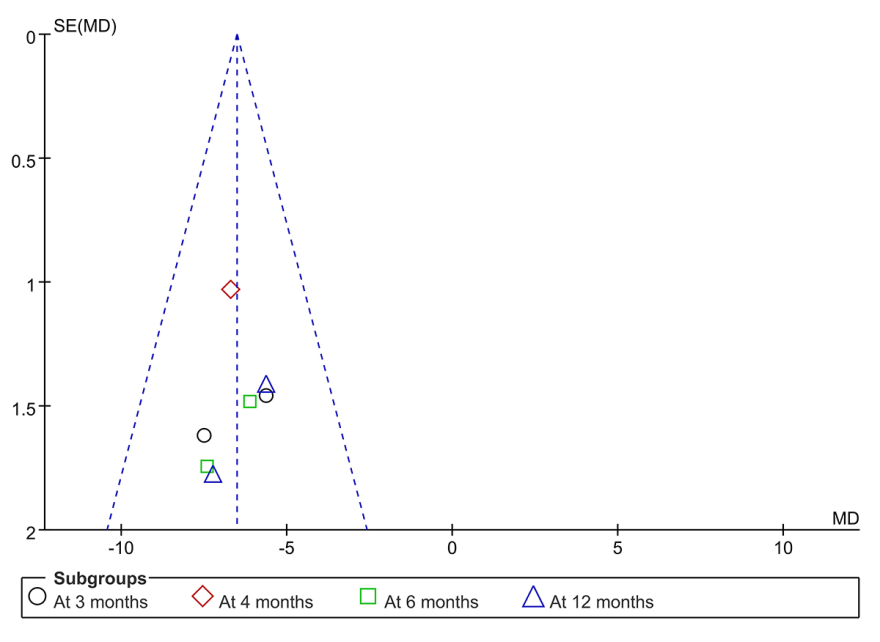


**Supplementary Figure 2** Funnel plot of pooled MDS-UPDRS III scores for the treated hemibody after unilateral FUS-STN. (A) Off-medication state at 1, 3, 4, 6, and 12 months; (B) On-medication state at 3, 4, 6, and 12 months.

(A)


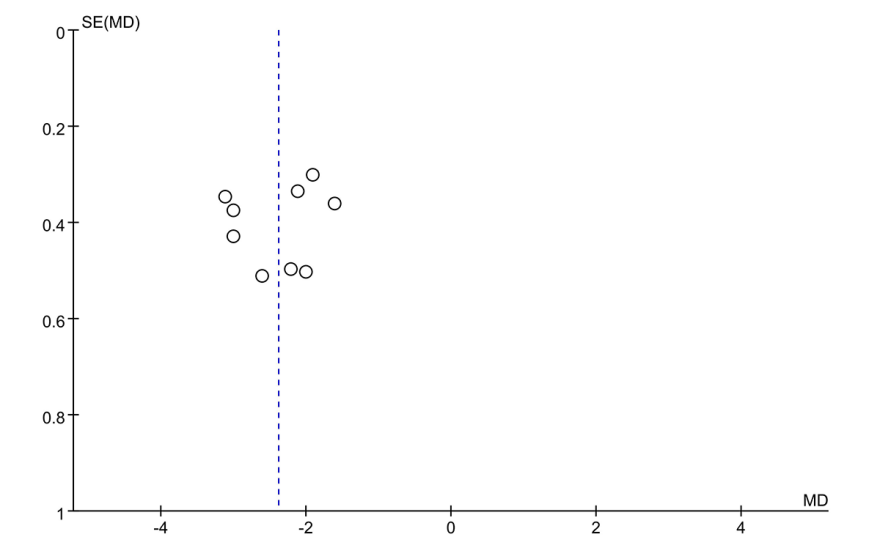


(B)


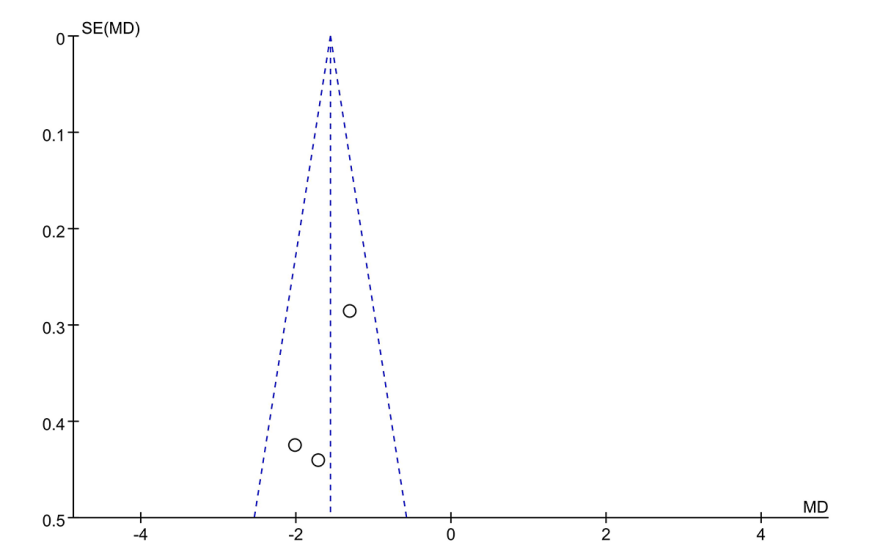


(C)


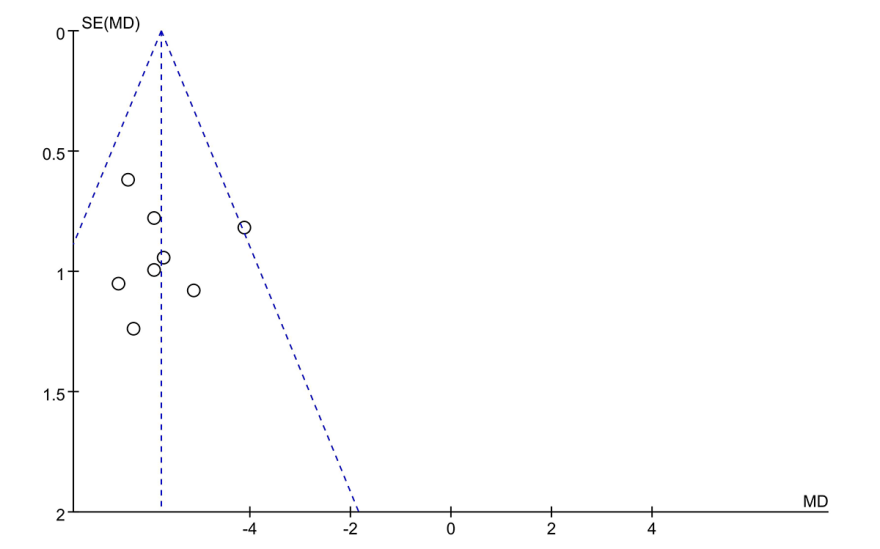


(D)


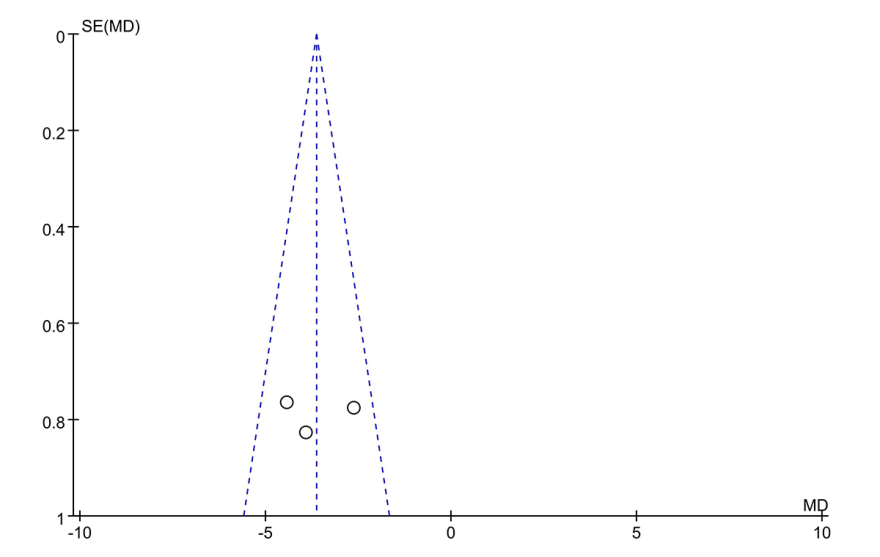


(E)


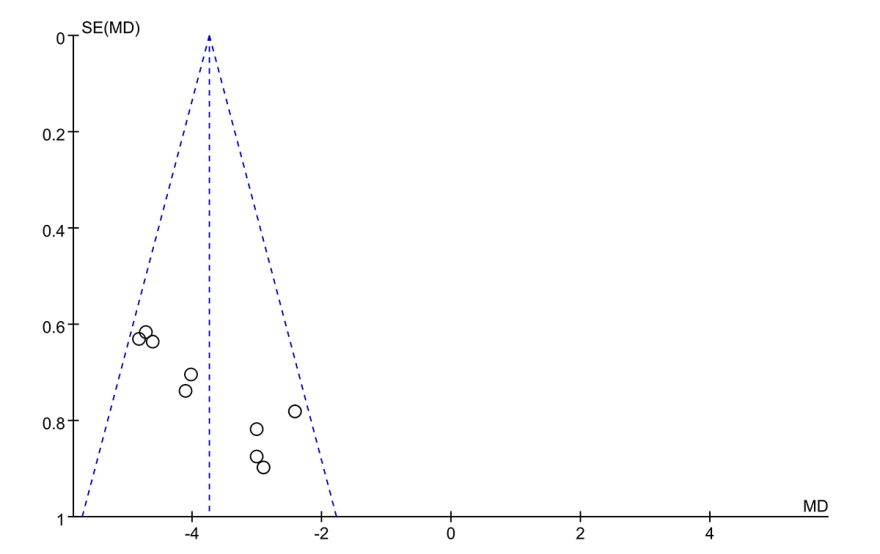


(F)


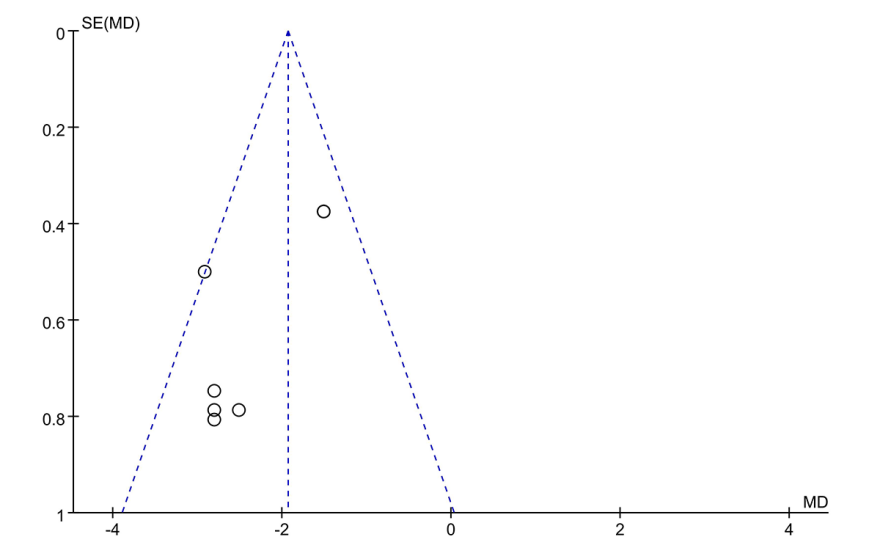


**Supplementary Figure 3** Funnel plot of pooled MDS-UPDRS III subitems scores for the treated hemibody following unilateral FUS-STN. (A) Rigidity in the off-medication state; (B) Rigidity in the on-medication state; (C) Bradykinesia in the off-medication state; (D) Bradykinesia in the on-medication state; (E) Tremor in the off-medication state; (F) Tremor in the on-medication state.


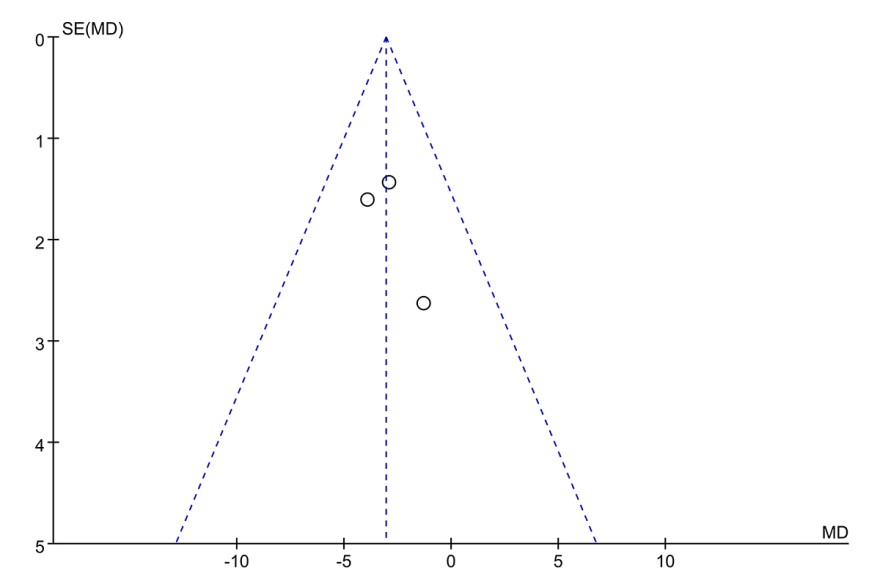


**Supplementary Figure 4** Funnel plot of pooled MDS-UPDRS II scores after unilateral FUS-STN


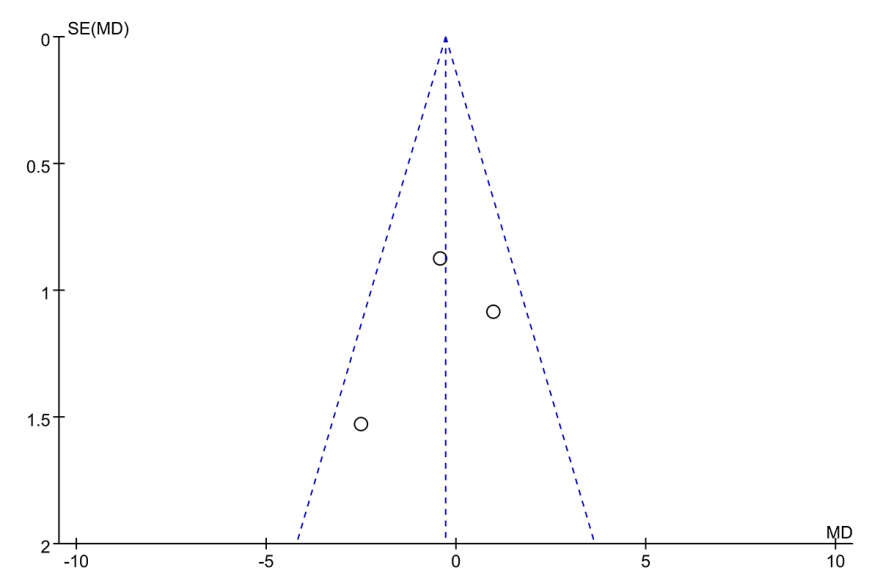


**Supplementary Figure 5** Funnel plot of pooled MDS-UPDRS IV scores after unilateral FUS-STN


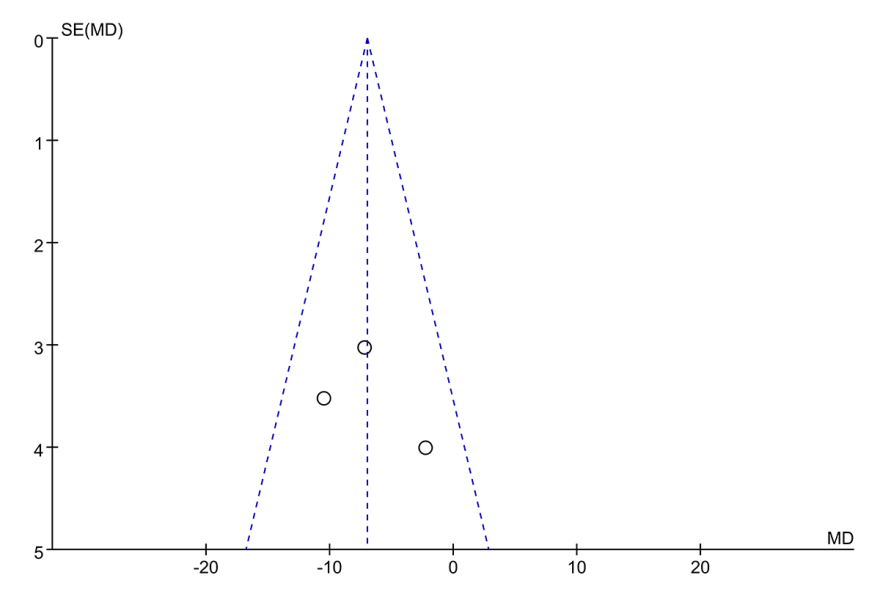


**Supplementary Figure 6** Funnel plot of pooled PDQ-39SI scores after unilateral FUS-STN


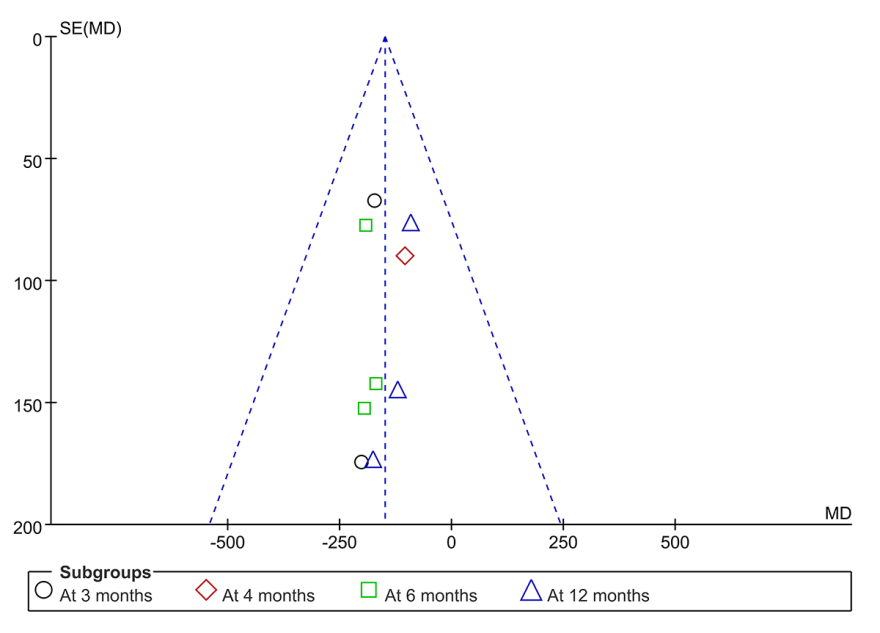


**Supplementary Figure 7** Funnel plot of pooled LEDD after unilateral FUS-STN at 3, 4, 6, and 12 months
